# Machine Learning Enabled Image Analysis of Time‐Temperature Sensing Colloidal Arrays

**DOI:** 10.1002/advs.202205512

**Published:** 2023-01-20

**Authors:** Marius Schöttle, Thomas Tran, Harald Oberhofer, Markus Retsch

**Affiliations:** ^1^ Department of Chemistry Physical Chemistry I University of Bayreuth 95447 Universitätsstr. 30 Bayreuth Germany; ^2^ Department of Physics Theoretical Physics VII University of Bayreuth Universitätsstr. 30 95447 Bayreuth Germany; ^3^ Bavarian Center for Battery Technology (BayBatt) University of Bayreuth Universitätsstr. 30 95447 Bayreuth Germany

**Keywords:** artificial neural network, film formation, photonic crystals, sensors, smartphone, structural colors

## Abstract

Smart, responsive materials are required in various advanced applications ranging from anti‐counterfeiting to autonomous sensing. Colloidal crystals are a versatile material class for optically based sensing applications owing to their photonic stopband. A careful combination of materials synthesis and colloidal mesostructure rendered such systems helpful in responding to stimuli such as gases, humidity, or temperature. Here, an approach is demonstrated to simultaneously and independently measure the time and temperature solely based on the inherent material properties of complex colloidal crystal mixtures. An array of colloidal crystals, each featuring unique film formation kinetics, is fabricated. Combined with machine learning‐enabled image analysis, the colloidal crystal arrays can autonomously record isothermal heating events — readout proceeds by acquiring photographs of the applied sensor using a standard smartphone camera. The concept shows how the progressing use of machine learning in materials science has the potential to allow non‐classical forms of data acquisition and evaluation. This can provide novel insights into multiparameter systems and simplify applications of novel materials.

## Graphical abstract

The optical response of multicomponent photonic crystals contains intricate information regarding a sample's thermal history. In this work, a reproducible sensor fabrication method coupled with fast data acquisition via digital photography is presented. Machine learning assisted evaluation allows smartphone‐based sensing of thermal events. Time and temperature can thereby be obtained independently and without specialized equipment.

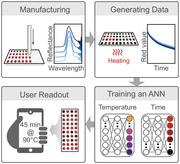

## 1 Introduction

Autonomous sensing has become increasingly important for various aspects of everyday life. For example, lifetime monitoring of batteries, food, and medicine requires tamper‐proof sensors independent of an external power supply.^[^ ^1^, ^2^, ^3^ ^]^ Color‐coded systems are advantageous since they allow user‐friendly readout.^[^ ^4^ ^]^ This prerequisite is often realized using the responsive photonic properties of nanostructured (often polymeric) materials.^[^ ^5^, ^6^ ^]^ These can react to external stimuli by changing the spacing, effective refractive index, or via loss of order.^[^ ^7^, ^8^, ^9^ ^]^ Beside the sensing of, e.g., pH‐value^[^ ^10^ ^]^ and (bio‐)analytes,^[^ ^11^ ^]^ temperature monitoring plays a key role in tracking degradation and spoilage.^[^ ^12^, ^13^ ^]^ Depending on the application, both reversible sensors and irreversible indicators have been shown.^[^ ^14^, ^15^ ^]^ More intricate systems can provide further information regarding the thermal history. Time–temperature integrators (TTIs) additionally provide temporal readout, which is highly relevant for establishing the safety of products.^[^ ^16^, ^17^, ^18^ ^]^ Often, this is achieved by controlling the kinetics of the deformation process in structured polymeric materials.^[^ ^19^ ^]^ A system shown by Lee et al. even allows the independent evaluation of time and temperature.^[^ ^20^ ^]^ This was possible by semi‐analytical characterization of the creep‐deformation process in polymeric inverse opals using local UV–vis spectroscopy. Recently, we showed a related material class: mixed colloidal crystals.^[^ ^21^ ^]^ These make use of adjustable dry‐sintering kinetics^[^ ^22^, ^23^ ^]^ and show great potential regarding evaluation using simple image analysis.

Sensing via RGB channels of images obtained with digital cameras greatly enhances the applicability compared to a spectral analysis. Examination using commercial, hand‐held devices rather than expensive (micro‐)spectrometers makes these appliances much more user‐friendly and more easily distributable. Research on such methods has been shown for, e.g., pH‐sensing^[^ ^24^ ^]^ and water‐content determination.^[^ ^25^ ^]^ Other materials for smartphone‐based temperature sensing allow readout via luminescence thermography.^[^ ^26^, ^27^, ^28^, ^29^ ^]^ Another path towards combining materials science with digital advancements is beginning to evolve in the form of machine learning.^[^ ^30^ ^]^ The application of these tools stretches from the prediction of optical properties^[^ ^31^ ^]^ to optimizing synthetic parameters to create the desired materials.^[^ ^32^ ^]^ For sensors, machine learning allows automated readout of complex, multiparameter systems that often cannot be described analytically. Examples comprise biomolecular sensing,^[^ ^33^ ^]^ ethanol content,^[^ ^34^ ^]^ and temperature.^[^ ^35^, ^36^ ^]^

Here, we introduce a TTI based on multicomponent colloidal crystals, using smartphone‐based image acquisition and machine learning analysis for the data evaluation. Four monodisperse polymer particle types are synthesized with varying glass transition temperatures to span a quaternary phase diagram. We use a fast, automated, and reproducible drop‐casting method to fabricate colloidal crystal arrays of mixed compositions. The composition correlates to the dry‐sintering kinetics and concomitantly to the loss of structural color. However, the quaternary particle system is too complex to allow an analytical description. Instead, we demonstrate that an artificial neural network can accurately measure our colloidal crystal arrays' time and temperature history. A system that initially is too intricate for conventional characterization is thereby made applicable for multiparameter sensing. Our analysis demonstrates a general approach to improve the sensing capabilities of well‐established photonic structures drastically. Due to the scalable fabrication process, the modular adjustment to other sensing tasks by a specific particle selection, and the user‐friendly, low‐tech characterization method, this TTI concept opens the pathway toward cheap multiparameter sensors.

## 2 Results and Discussion

We aim to fabricate a sensor enabling a simple readout of two independent parameters: time and temperature. One main difficulty, thereby, is designing a system that is complex enough to over‐determine the parameter space yet remains feasible to analyze. The concept presented here is based on an array of polymer colloidal crystals (CCs). The first step, therefore, is the realization of a suitable self‐assembly process. Prerequisites for sample preparation are site selectivity, reproducibility, automation, and a fast preparation rate. Consequently, we apply a combination of array‐printing and drop‐casting that meets these criteria and additionally is scalable, resource‐efficient, and non‐toxic.

**Figure 1:**
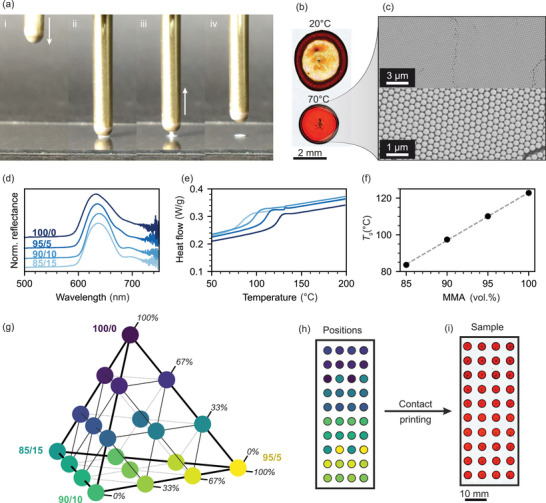
Fabrication process of multi‐spot colloidal crystal sensors. a) Snapshots of the array‐printing procedure, showing the (i) advancing, loaded tip, (ii,iii) the tip in contact with the substrate, and (iv) the receding pin. b) Microscopy images of spots prepared at different substrate temperatures, elucidating the importance of accelerated evaporation during self‐assembly. c) SEM images of the colloidal crystal shown in panel (b). d) UV–vis reflectance spectra of four spots prepared from copolymer particles of identical size but different comonomer compositions. e) DSC heating curves of the four different copolymers. f) Glass transition temperatures obtained from panel (e), showing a linear dependence regarding the comonomer composition. g) Quaternary phase diagram of all particle mixtures obtained from mixing the four different particle types. h) Positions of these mixtures on the substrates. i) Microscopy image of a substrate prepared via the array‐printing of the mixed particle suspensions shown in panels (g) and (h).

A spring‐loaded pin with a hydrophilic, round tip is dipped into a particle suspension that adheres via wetting. When brought into contact with a glass substrate, a defined dispersion volume is deposited and subsequently forms a CC via evaporative self‐assembly (**Figure** **1**a). The interplay of capillary and Marangoni flow in these sessile droplets at room temperature results in a pronounced coffee‐stain effect (Figure 1b).^[^ ^37^ ^]^ Structural colors appear faint and far from homogeneous, and the droplet itself shows an irregular shape. When heating the substrate to 70 °C, the interactions favor a homogeneous layer of particles, facilitated by the formation of a “milk‐skin”‐like particle layer during the accelerated evaporation.^[^ ^38^ ^]^ Additionally, evaporation occurs at the edges immediately after contact, forcing the assembly to occur in a well‐defined circular area. This greatly enhances the reproducibility and, thereby, the readability of the sensor during the analysis described later. Scanning electron microscopy (SEM) images of the surface show large domain sizes of densely packed, monodisperse particles, corroborating the vivid structural colors observed via light microscopy (Figure 1c). Another significant feature of this process is efficiency, as almost none of the suspension is wasted. Therefore, a given laboratory‐scale batch of particles (typically a few 100 mL with 5 wt.% particle concentration) can theoretically be used to prepare several thousand samples.

Having established a robust array fabrication method, we now present the cornerstones of the particulate system. The polymer latex particles used in this work consist of random copolymers of methyl methacrylate (MMA) and *n*‐butyl acrylate (nBA). Four different particle types are prepared with varying comonomer volume ratios between 85:15 and 100:0 while maintaining a consistent particle diameter of 320 ± 5 nm. Self‐assembly of all four particle types and subsequent UV–vis spectroscopy (Figure 1d) show an optical stop band at 635 ± 3 nm in each case. Both the assembly behavior and the periodicity of the resulting nanostructure are thereby proven to be uniform. The differences between the four particle types are elucidated via differential scanning calorimetry (DSC). Heating curves show the glass transition temperature (*T*_g_) shifting towards higher temperatures when increasing the MMA content (Figure 1e). This dependency of *T*_g_ and comonomer composition is linear (Figure 1f).

The key aspects of these building blocks are the same size and surface chemistry of the particles with different thermal properties. This allows the fabrication of multicomponent yet crystalline nanostructures from mixed particle suspensions. Depending on the number of components in an ensemble, the film formation process can be tailored to a specific temperature range. The thermal parameter space, we apply for sensing, can be elucidated in a quaternary phase diagram (Figure 1g) showing all utilized particle mixtures. The automated array‐printing setup facilitates the realization of this large parameter space. We, therefore, drop‐cast a total of 20 different particle mixtures onto defined positions on a glass substrate (Figure 1h). Two spots are prepared with each composition to introduce some redundancy and improve later readout. The setup allows reproducible fabrication of samples with circular spots of CCs, all with the same vivid, red structural coloration due to a consistent periodicity, geometry, and effective refractive index (Figure 1i). Differences can later be observed at elevated temperatures, where the thermal response of each composition is tracked.

**Figure 2:**
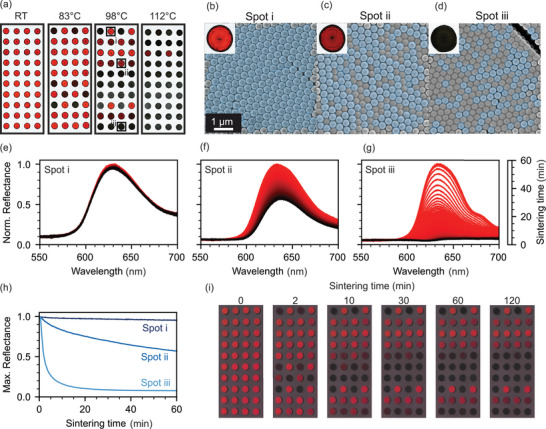
Thermal response of the sensors. a) Light microscopy images of samples held at different temperatures for 120 min. The width of each image is 27 mm. b–d) SEM images of the spots indicated in panel (a). The blue overlay shows intact, non‐sintered particles. e–g) In situ UV–vis spectra of equivalent spots during the sintering process at 98 °C showing the gradual stop‐band degradation. These, however, have to be measured consecutively. h) Time‐dependent decrease of the normalized stop‐band intensity. i) Photographs taken in situ of a sample during the sintering process at 98 °C with a smartphone camera.

The question now is how to characterize such a sample appropriately. Classic laboratory characterization methods can be divided into two groups: 1) Methods that exhaustively cover the entire sample but can only be measured ex situ. 2) In situ methods that are, however, limited to one spot at a time. An example of ex situ characterization is scanning light and electron microscopy (**Figure** **2**a–d). A pristine sample (RT), as well as three samples subjected to isothermal sintering at different temperatures between 83 and 112 °C for 120 min are shown. Depending on the thermal history, specific CCs remain (nearly) pristine, while others show various degrees of discoloration. We examine three representative positions post‐sintering via SEM to corroborate the expected structural change (Figure 2b–d). The respective CCs consist of particles with 90%, 95%, and 100% MMA and show compositions of 0:1:2 (spot i), 1:1:1 (spot ii), and 2:1:0 (spot iii). When the CC consists of only high‐*T*_g_ particles (spot i), the structure remains intact after heating (blue‐shaded particles). When only the minority phase is affected by the temperature increase, and these particles deform (spot ii), an interconnected nanostructure of periodically arranged particles remains. As the temperature persists, these voids are slowly filled by the creeping polymer. The overall refractive index contrast between spheres and voids is concomitantly reduced, and the saturation diminishes. If the majority of particles are heated above their *T*_g_ (spot iii), only small islands remain and (nearly) all symmetry and periodicity are lost. No discernable color remains.

Complementary to this ex situ evaluation, in situ UV–vis spectroscopy provides temporal information regarding the sintering process. Three spots are measured, one after the other (Figure 2e–g). The spectrum of spot i shows little to no change during 60 min at an elevated temperature. Spot ii, however, shows a slow and consistent degradation of the stop‐band to approximately half of its previous reflectance. Spot iii shows a fast response, with almost complete loss of any indication of a photonic stop‐band during the first 10–15 min. Quantifying the time‐dependent UV–vis spectra is possible, e.g., in the form of the normalized stop‐band intensity (Figure 2h). However, it is unfeasible to perform this measurement at all 40 spots at once. Spectroscopic methods, therefore, fail to provide a holistic evaluation of the sensor's response to thermal events. Additionally, while similar optical studies of inverse polymeric opals have been conducted by applying, e.g., the Kelvin–Voigt model and WLF theory,^[^ ^20^ ^]^ our system is difficult to be studied (semi‐)analytically.^[^ ^21^ ^]^ Sintering of particulate systems, in general, is a multi‐step process,^[^ ^39^, ^40^ ^]^ and the binary and ternary mixtures increase this intricacy. Besides the polymer and particle composition, the surface chemistry may influence the film formation kinetics. All this renders an analytical description of the film formation increasingly difficult.

Machine learning lends itself as a prime candidate for evaluating the behavior of our sensors. It can describe nonlinear behavior without requiring extensive physical modeling. Instead, a prerequisite for machine learning is a large amount of data. We acquire the necessary data by capturing the time‐dependent optical response of the sensor using a smartphone camera. This unconventional yet convenient method has the additional benefit of being widely applicable and providing a user‐friendly and non‐expert evaluation. Capturing the response with a smartphone combines the time‐resolution of the in‐situ UV–vis spectroscopy with the ability to measure the entire sensor of scanning microscopy (Figure 2i).

**Figure 3:**
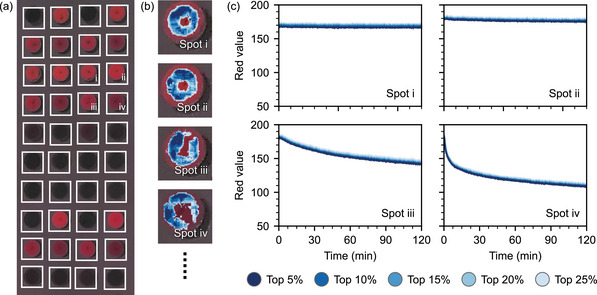
Preprocessing of the image data for the neural network. The shown image was taken after heating the sample for 80 min at 98 °C. a) The digital image of the sensor is divided into 40 sub‐images containing one spot each. b) For each sub‐image, the pixels with the highest red value are used for further evaluation. c) The mean red values follow the same trend as the stop‐band decay.

An evaluation of the full images is computationally expensive and includes many pixels of the substrate background that contain no relevant information. Furthermore, slight differences between spot sizes will complicate the training process. We, therefore, determine the mean red value of each spot by dividing every image into 40 sub‐images containing one spot each (Figue **3**a). We use the mean brightness of the 5%, 10%, 15% , 20%, and 25% of pixels with the highest red value for the evaluation (Figure 3b). For each substrate, 40 spots with five mean values each correspond to 200 inputs for a given image. Compared to the RGB images with a size of 420×1060 pixels, the number of inputs is reduced by a factor of ≈6700, significantly speeding up computations. The mean red value of each spot (Figure 3c) changes similarly to the stop‐band intensity shown in Figure 2h. Spots i and ii both show little to no change during 120 min of isothermal heating. Also, the absolute red value of the two spots is nearly identical, corroborating the homogeneity and structural integrity of the CCs. The mean red value of spot iii decreases continuously throughout the measurement, while spot iv shows a fast degradation during the first 10 min. Combining these different response types to an elevated temperature is important for making a reasonable readout possible. For comparison, we also show analogue plots for samples measured 5 K above and below this temperature (Figure S1, Supporting Information). The influence of the change in temperature on the sintering kinetics is clearly visible in each decay curve. Therefore, we conclude that thermal and temporal information is hidden in the 200 inputs and continue to establish a model capable of deciphering the results.

**Figure 4:**
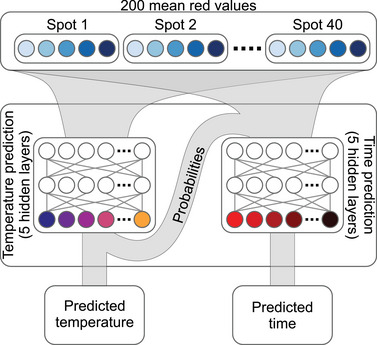
Artificial neural network architecture. The model consists of two parts. The first half predicts the temperature by using only the mean red values. The second half predicts the heating time, with the inputs being the mean red values and the temperature probabilities.

We use artificial neural networks (ANNs) with ten hidden layers to predict the time and temperature of a single image. The design idea for our ANN is to model the distinct sintering kinetics of each particle composition at each temperature. Therefore, the model consists of two parts (**Figure** **4**). First, the model estimates the probability of an image being taken at a specific temperature by detecting the pattern of spots with no, little, and high red intensities. The resulting probability density and the mean red values are the inputs for the time prediction layer. Finally, the model reports the most probable temperature and predicts the time as a continuous variable. A detailed description of the network architecture and training procedure is in the Experimental Section.

We trained the ANN with nine different temperatures between 100 and 140 °C. Here, we report the hot plate set point as the temperature for better readability. The set point is slightly higher than the actual sensor temperature (Figure S2, Supporting Information). At each temperature, we measured eight samples for 2 h at intervals of 5 s, corresponding to >94000 training images. Supervised training optimizes the model parameters, and after 20 training epochs, the model assigns 96.7% of training images to the correct temperature (Figure S3a, Supporting Information). The predicted time also correlates very well with the measured time. More than 80% of training inputs deviate <10 min from the correct value (Figure S3b, Supporting Information). We notice that the wrong assignment of temperatures occurs primarily at short times and that the incorrectly predicted temperature is directly below the correct temperature (Figure S3c, Supporting Information).

**Figure 5:**
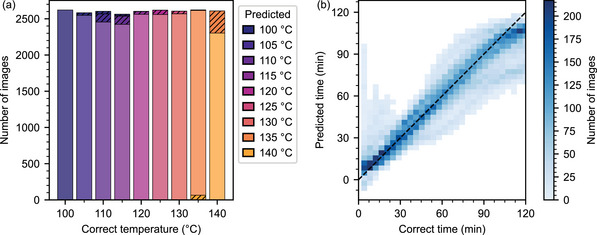
Prediction results for two samples per temperature, corresponding to >23000 validation images. a) Correlation of correct and predicted temperatures. Incorrect predictions (hatched areas) are minimal and mostly show a deviation of only 5 K from the correct value. Underestimations are shown at the top, and overestimations at the bottom. b) Correlation between correct and predicted time values.

Next, we investigate the generalization of our ANN by predicting the time and temperature for two validation samples per temperature. Our model has never seen these samples before and is unaware of the correct values. We can validate our system over the whole time–temperature regime because both the sensor creation and the sensor evaluation are automated. In total, the validation set consists of >23000 images. As shown in **Figure** **5**, the resulting predictions resemble the training results well. Temperature predictions are correct for the most part (96.4%). If images are mislabeled, the temperature error is mainly only 5 K (3.3%). Concerning the time, the majority of the predictions closely follow the correct value (Figure 5b). However, some predictions deviate from expectations. The large number of validation images allows us to investigate these deviations in more detail by grouping the time predictions by the correct temperature.

**Figure 6:**
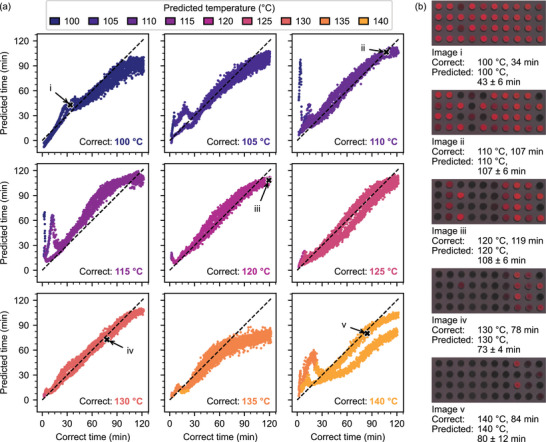
Detailed prediction results for the validation images. a) Predictions for the first seven temperatures are very close to the true values. At higher temperatures, the time predictions begin to deviate. b) Input images of five validation points from (a) with their corresponding predictions.

**Figure** **6**a shows how the prediction quality varies with the correct sensor temperature. Each point in the graphs corresponds to one validation image. For most temperatures, no difference between the two used validation samples is visible, demonstrating that both the creation and evaluation of our sensors are highly reproducible. While predictions at temperatures below 135 °C are very accurate, some images at the highest temperatures show incorrectly predicted time values. As the same phenomenon occurs in the training data (although less pronounced), this is not a generalization issue but a limitation of the applied system itself.

**Figure 7:**
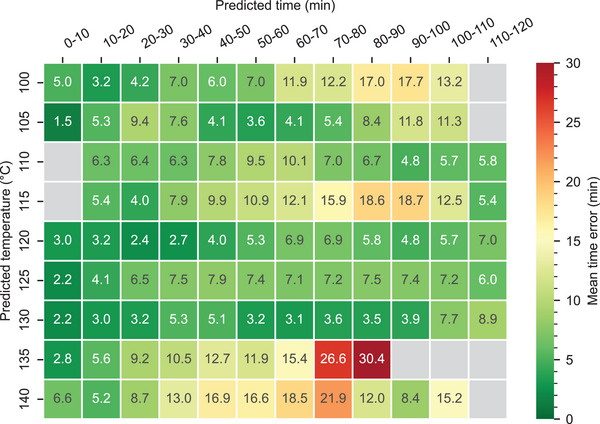
Mean errors between predicted and correct time. The validation over the whole time–temperature regime allows estimating uncertainties precisely. Lower temperatures show a smaller deviation from the correct value. For high predicted temperatures, the uncertainty increases.

Without a large amount of validation data, it is impossible to identify the prediction capabilities in the distinct areas shown above. Previous publications about TTIs validated their system with a small number of validation samples,^[^ ^15^, ^18^, ^20^, ^21^ ^]^ thus, not covering the whole time–temperature regime. Our large amount of validation data allows us to state individual uncertainties for each pair of predicted temperature and time (**Figure** **7**). The mean absolute difference between the predicted and the measured time is generally below 10 min for temperatures below 135 °C. For high temperatures, the uncertainty is larger. These individual errors can be used as an output for the end user. Examples of single images as recorded by a potential user are shown in Figure 6b.

Further examples are in Figures S4–S6 (Supporting Information), showing how our integrators behave with multiple temperature steps. As expected, if the sensors cool down between two isothermal heating steps, the predicted time is the sum of the two heating durations (Figure S4, Supporting Information). If multiple heating events in the temperature range of the sensors occur, the prediction refers to the higher temperature and further heating at lower temperatures does not affect the readout (Figure S5, Supporting Information). For small temperature differences, a slight overestimation of the time is possible (Figure S6, Supporting Information). Consequently, this type of TTI sensor is most suitable and applicable for the recording of the highest temperature events, which are, in many cases, the most relevant ones to judge on safety or spoilage issues.

The evaluation of a single photograph takes <1 s and is based solely on an image taken by a smartphone camera. No knowledge of photonic systems or the underlying physical processes is necessary to utilize our system. The software will immediately predict the time and temperature of the photographed sensor and state the corresponding uncertainty. Consequently, non‐specialists can employ our system effortlessly. This concept can conceivably be adjusted to adapt the prerequisites to various applications. The time and temperature ranges that can be determined are related only to the thermal properties of the respective polymer particles. Changing the glass transition temperature of these can easily be done by varying the monomer composition. Alternatively, high‐temperature applications can be made possible by adding inorganic components such as silica colloids to the phase diagram. Since the process is irreversible, tamper‐proof monitoring of goods such as food or batteries becomes a simple process. Further advancements can be readily implemented by miniaturizing the colloidal arrays down to the image resolution limit of commercial cameras. Thereby, an even larger number of CC spots and, consequently, particle mixtures could be examined at once. Increasing the number of CC spots will also provide flexibility to include particle mixtures with different stop‐bands allowing for a multi‐color analysis specific to certain temperature ranges.

## 3 Conclusion

We established a concept that applies a combinatorial approach to add significant functionality to the well‐known material class of polymer colloidal crystals. Mixed photonic systems described by a quaternary phase diagram were assembled using a scalable and efficient array‐printing method. This allowed us to examine the thermal response of numerous samples, which formed a solid training set for our measurement evaluation. The sensor state can be optically read out by digital photography using a standard smartphone. The evaluation was performed using an artificial neural network. Using only the photograph of a sample subjected to isothermal heating, the model correctly predicts time and temperature independently. Our concept can be readily transferred to specific sensing applications comprising photonic structures and integrating sensing capabilities. The case demonstrated here is particularly simple owing to the robust array fabrication procedure and the optical readout, which make this sensor useful for non‐expert users. Overall, we showed how the combination of materials chemistry and advanced computational methods are starting to enable a multiparametric analysis from complex colloidal systems.

## 4 Experimental Section

### Materials

Methyl methacrylate (MMA), *n*‐butyl acrylate (nBA), 3‐styrenesulfonic acid sodium salt hydrate (NaSS, 99%), and potassium persulfate (KPS, 99%) were obtained from Sigma–Aldrich. Before further use, both MMA and nBA were destabilized over Alox B. Water of MilliQ quality was used throughout all experiments. Glass substrates were cleaned via sonication in an aqueous 2 vol.% Helmanex III solution and in ethanol.

### Particle Synthesis

Monodisperse particles were prepared via a surfactant‐free emulsion polymerization. 240 mL water were heated to 80 °C and degassed in a 250 mL three‐necked flask for 75 min. While stirring at 600 rpm, 19 mL of the respective monomer mixture were added, together with 10 mg NaSS dissolved in 5 mL water. After 5 min, the polymerization was initiated by adding 200 mg KPS dissolved in 5 mL water. The reaction was left to proceed overnight and terminated by exposure to ambient oxygen. The different particle dispersions were each filtered over a 125 µm mesh and otherwise used directly for preparing the binary and ternary mixtures. The concentration of all dispersions was 5.7 ± 0.1 wt.%.

### Self‐Assembly via Array‐Printing

The printing procedure was fully automated using an XYZ stage to ensure full reproducibility. A clean glass substrate was placed on a hot‐plate set to 70 °C. A spring‐loaded, rounded brass pin with a diameter of 5 mm was dipped into a dispersion and then brought in contact with the substrate for a duration of 1 s. The pin was then mechanically cleaned in a water bath and dried with a non‐woven fabric. The process then repeated with the next dispersion.

### Characterization Methods

Microscopy images were obtained using a laser scanning confocal microscope (Olympus, OLS5000) with a white light source as well as a 405 nm laser with a 5×‐magnification lens and stitching of 7×18 images.

Scanning electron microscopy images were obtained with a Zeiss Leo 1530 (Carl Zeiss AG, Germany) at an operating voltage of 1 kV and both in‐lens as well as secondary electron detection after sputtering of 2 nm platinum. Images, where a false‐colored overlay was applied, are shown in their original form in Figure S7 (Supporting Information).

UV–vis spectra of drop‐cast suspensions were measured on an Olympus IX71 inverted microscope with a 10× lens in reflection geometry and a halogen light source. An OceanOptics USB4000 spectrometer was coupled via fiber optics. In situ measurements were conducted using an Instec HCS622HV heating stage with a silver heating block set to 110 °C. Samples were attached to the stage using double‐sided carbon tape, and the sample was heated to 98 ± 3 °C. Spectra were obtained at intervals of 2 s.

Differential scanning calorimetry was conducted using a TA Instruments Discovery DSC 2500. The second of two heating cycles was used for the evaluation. Samples were measured between 20 and 200 °C at 10 K min^−1^ and in a nitrogen atmosphere.

The hydrodynamic diameter was measured using diluted dispersions with a Zetasizer (Malvern) with 175° backscattering geometry.

### Image Acquisition and Feature Extraction

Each sample was placed on a black‐coated hot plate (PZ 28‐2, Harry Gestigkeit GmbH). A full‐spectrum lamp (Walimex pro LED Niova 600 Plus Daylight) with a light diffuser illuminated the sample at an angle of 10° and a distance of 30 cm. A smartphone (Fairphone 3+) took photographs (ISO 100, 1/10647 s exposure time) of the sample at an angle of 10° and a distance of 10 cm every 5 s, stored in the WebP format. The full 3000×4000 pixel images were cut into 40 squares of 75×75 pixels at pre‐defined positions. The cropped images are available online.^[^ ^41^ ^]^ For each square, the 5%, 10%, 15%, 20%, and 25% pixels with the highest red value were used to determine five distinct mean values used as the input for the ANN. Each input vector of length 200 was standardized by z‐score normalization using the mean and standard deviation of the training set.

### ANN Architecture

PyTorch^[^ ^42^ ^]^ was used for the network creation and we made the code available online.^[^ ^43^ ^]^ To choose a suitable model structure, different architectures were compared. Details are in the Supporting Information. The final machine learning approach encompasses two almost identical, sequential models for temperature and time. They consist of an initial batch normalization layer and five hidden, linear layers each. The hidden layers have node sizes of 8192, 2048, 2048, 2048, and 512, respectively. Each hidden layer uses a leaky ReLU function^[^ ^44^ ^]^ as its activation. After the final hidden layer, a dropout layer with a dropout probability of 50% was introduced to improve generalization. For the temperature module, the output layer was a softmax function creating a probability density for the nine temperature categories. For the time module, the output layer was a final linear layer of size one.

### Training Process

The time series images of eight samples per temperature were labeled and used for training. The initial 2 min of each sample were discarded due to temperature equilibration (Figure S2, Supporting Information). Prior to training, the time labels were scaled by min–max normalization with a minimum time of 2 min and a maximum of 122 min. Stochastic gradient descent was employed. Different hyperparameters were tested (Table S2, Supporting Information). The final model was trained with a batch size of 32, a learning rate of 5×10^−4^, a Nesterov momentum of 0.9 and a weight decay of 1×10^−3^. The loss function is the sum of the cross‐entropy loss for the temperature prediction and the mean squared error for the time predictions. The training concluded after 20 epochs.

### Statistical Analysis

The errors indicated for the predicted times are mean deviations between the correct and predicted times of the validation data. To determine those, predictions were grouped into the temperature and time bins shown in Figure 7. For each bin, the mean absolute difference between the correct and predicted value is shown. The training set consists of 94032 and the validation set of 23433 images. Preprocessing of the photographs is explained in the subsection “Image Acquisition and Feature Extraction” . The data and software are available online.^[^ ^41^, ^43^ ^]^

## Conflict of Interest

The authors declare no conflict of interest.

## Supporting information

Supporting Information

## Data Availability

The data that support the findings of this study are available from the corresponding author upon reasonable request.
