# Supplementary material for: Machine Learning Enabled Image Analysis of Time‐Temperature Sensing Colloidal Arrays
Source: Adv Sci (Weinh). 2023 Jan 20;10(8):2205512. doi: 10.1002/advs.202205512 (PMC10015860; doi:10.1002/advs.202205512)
Supplement: Supplementary file 1 — Supporting Information [file ADVS-10-2205512-s001.pdf]

Supporting Information: Machine learning enabled image analysis of time-temperature sensing colloidal arrays

*Marius Schöttle Thomas Tran Harald Oberhofer Markus Retsch**

M. Schöttle, T. Tran, Prof. M. Retsch
Department of Chemistry, University of Bayreuth, Universitätsstr. 30, 95447 Bayreuth, Germany
Email Address: markus.retsch@uni-bayreuth.de

Prof. H. Oberhofer
Department of Physics, Theoretical Physics VII, University of Bayreuth, Universitätsstr. 30, 95447 Bayreuth, Germany

Prof. H. Oberhofer, Prof. M. Retsch
Bavarian Center for Battery Technology (BayBatt), University of Bayreuth, Universitätsstr. 30, 95447 Bayreuth, Germany

1 ANN Architecture

We compared five different model and chose the best one to be our final architecture (Tab. S1).

Full images The input for this model are the RGB images of our sensors instead of the average red values. Multiple convolutional layers preceed the fully connected layers.

Single model Instead of splitting the model into parts dedicated to the temperature and time prediction, we use the outputs of the second to last layer to one independent layer for time and temperature, each.

Small model The number of nodes of this model is reduced to 1/16th of the final model.

Medium model The model architecture described in the main text.

Large model The number of nodes of this model is four times larger than that of the final model.

Table S1: Comparison between different network architectures. The final model is highlighted.

Model	Traning loss	Validation loss
Full images	1.38	1.62
Single model	1.45	1.48
Small model	1.52	1.54
Medium model	1.41	1.42
Large model	1.43	1.49

Table S2: Comparison between different training parameters. Shaded parameters are used to train our model.

Batch size	Learning rate	Momentum	Weight decay	Traning loss	Validation loss
32	5×10^{-4}	0.7	1×10^{-3}	1.56	1.54
32	5×10^{-4}	0.98	1×10^{-3}	1.44	1.49
32	5×10^{-4}	0.9	1×10^{-4}	1.45	1.42
32	5×10^{-4}	0.9	5×10^{-3}	1.53	1.50
16	5×10^{-4}	0.9	1×10^{-3}	1.46	1.44
64	5×10^{-4}	0.9	1×10^{-3}	1.50	1.49
32	1×10^{-4}	0.9	1×10^{-3}	1.61	1.54
32	5×10^{-4}	0.9	1×10^{-3}	1.41	1.42
32	1×10^{-3}	0.9	1×10^{-3}	1.44	1.44

2 Supporting Figures

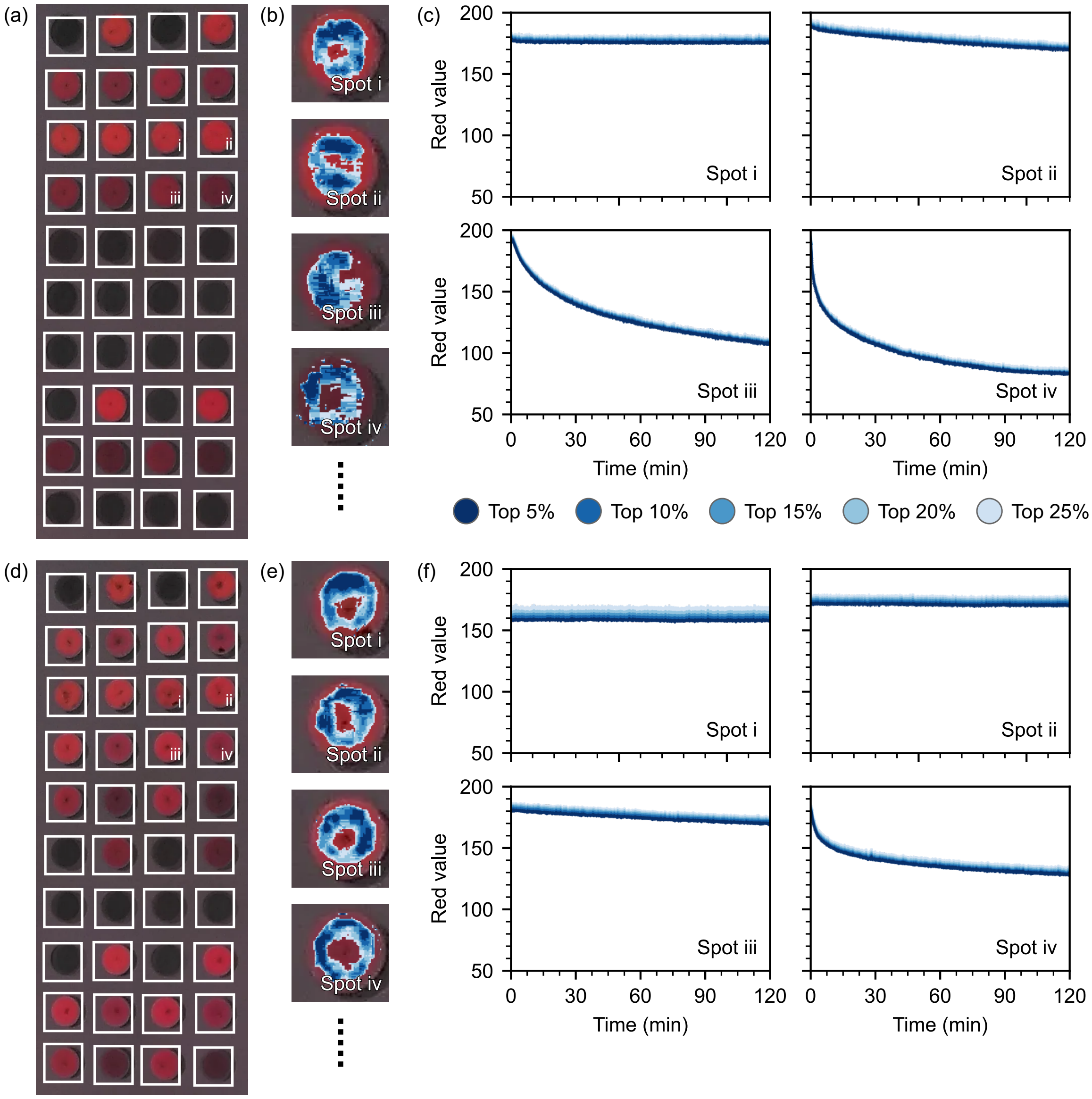

Figure S1: Detection of mean red values for samples at different temperatures. (a–c) 125 °C, (d–f) 115 °C.

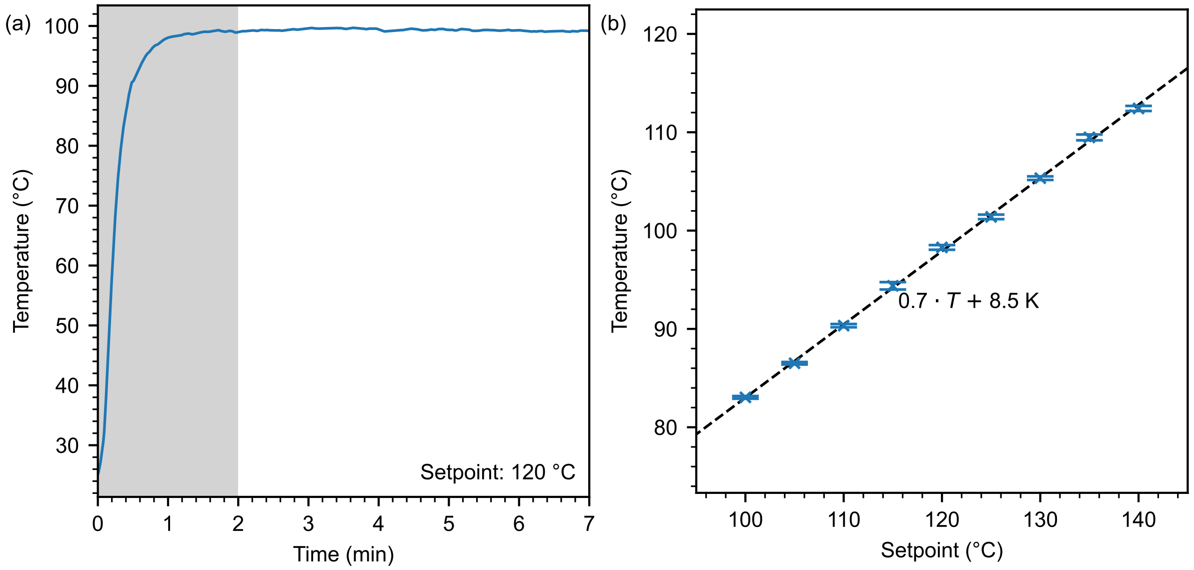

Figure S2: Sample temperature on the hotplate. The true temperature was measured by a Pt-100 placed on top of a glass substrate. (a) During the first two minutes (shaded area), the sample reaches thermal equilibrium. (b) Due to the experimental setup, the sample temperature is lower than the setpoint of the hotplate.

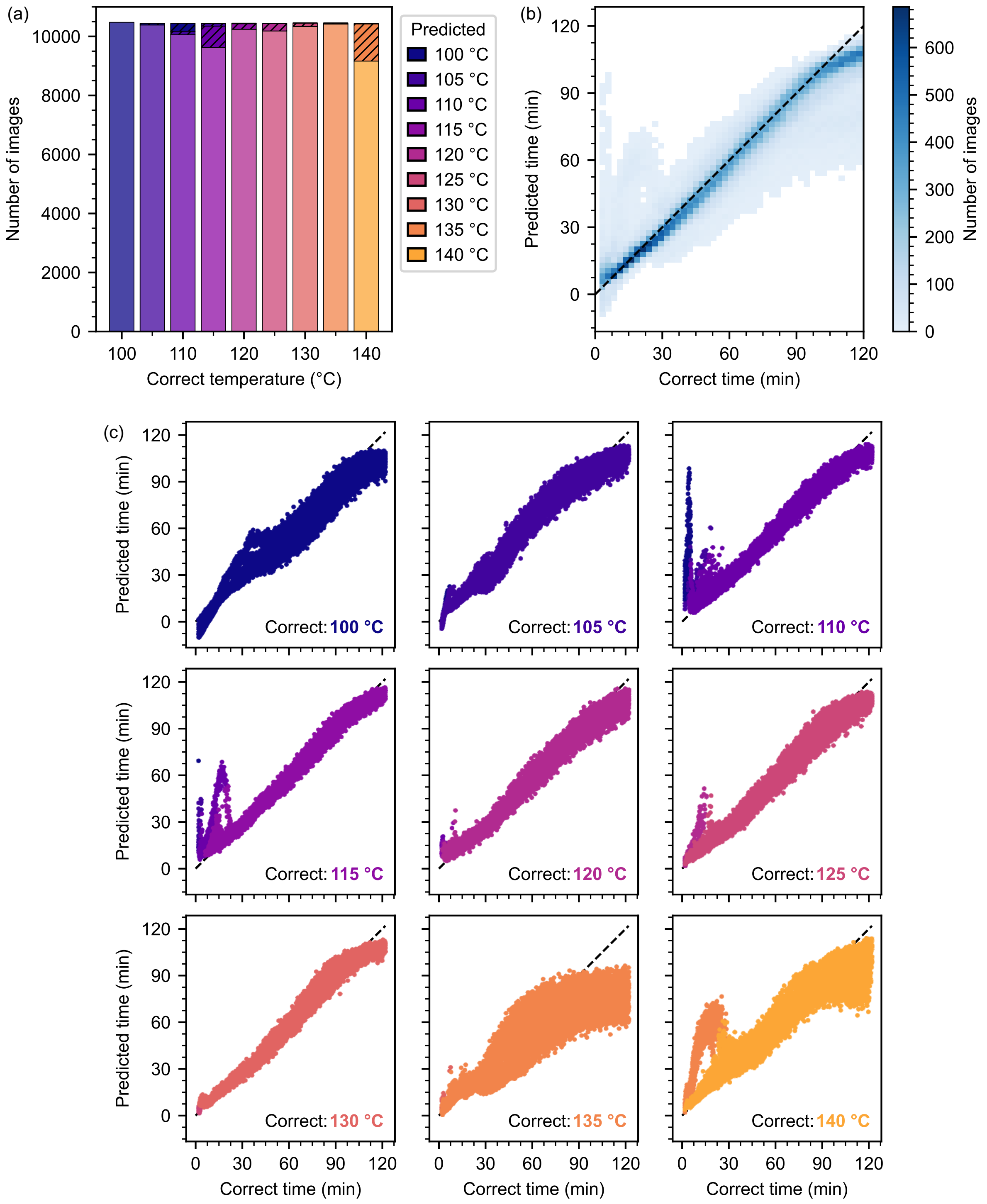

Figure S3: Prediction results for the training data. (a) Temperature prediction. (b) Correlation between predicted and correct time. (c) Detailed view of time and temperature prediction.

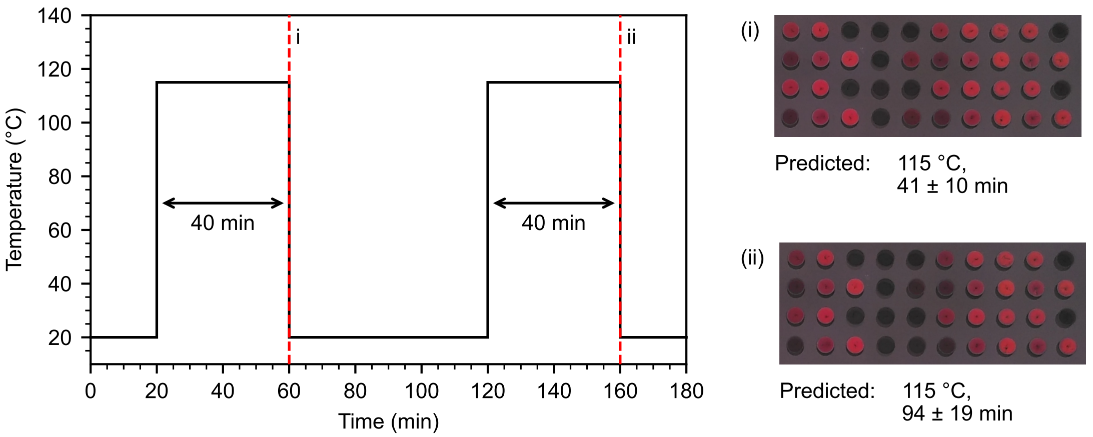

Figure S4: Prediction results with complete cooldown. The graph shows the applied temperature profile. Evaluation of the sensor after both heating steps shows is as expected. The sensors integrate the total time at the elevated temperature.

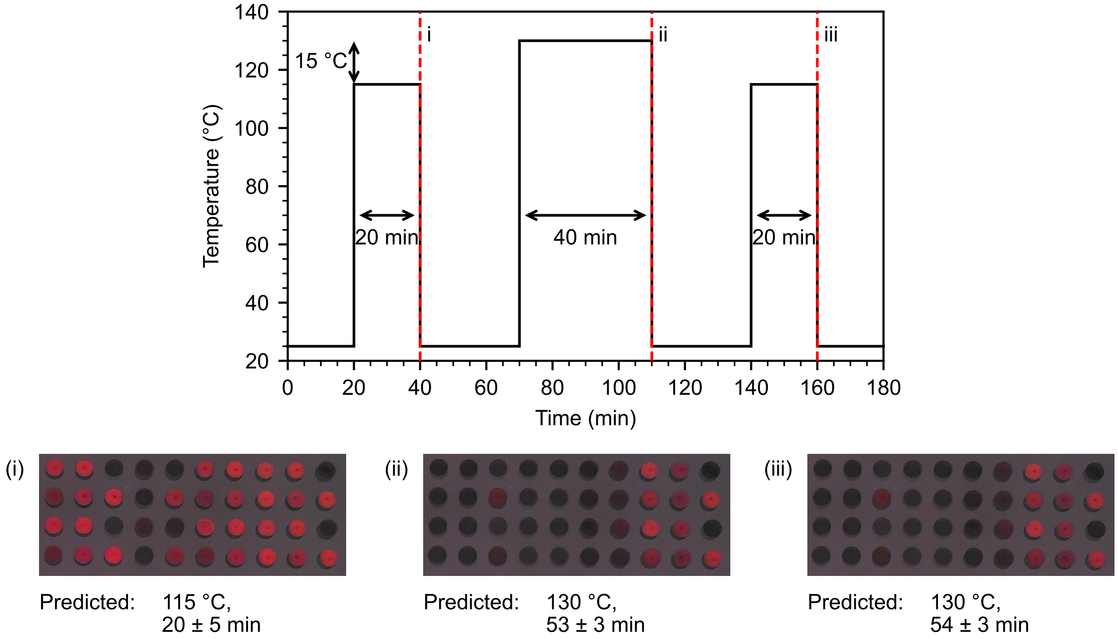

Figure S5: Multiple heating events inside the operating range of the sensor. The sensor reports the highest detected temperature and the corresponding heating time.

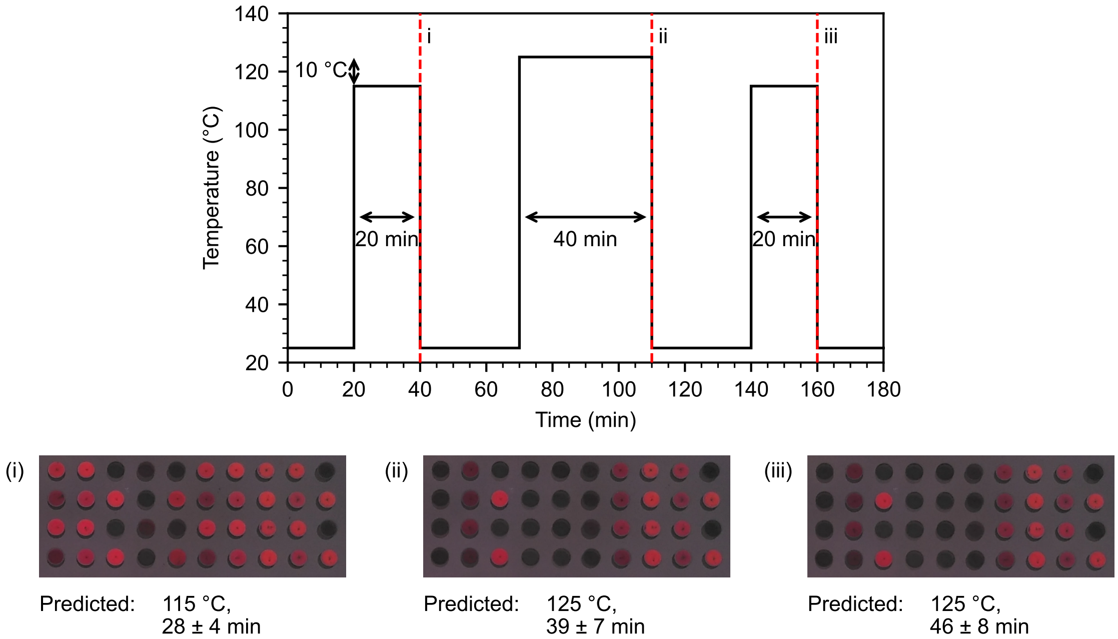

Figure S6: Multiple heating steps with a small temperature difference. Our sensor shows the expected result after the first and second heating step. After the third heating step, the predicted time slightly increases.

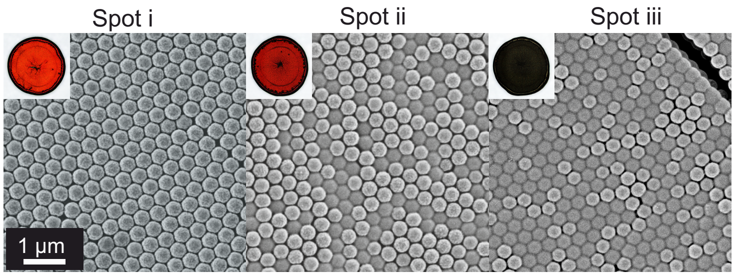

Figure S7: Original SEM images of partially sintered colloidal crystals shown in Fig 2b–d.
